# Identifying trajectories of fatigue in patients with primary mitochondrial disease due to the m.3243A > G variant

**DOI:** 10.1002/jimd.12546

**Published:** 2022-08-24

**Authors:** Inge‐Lot Klein, Christianne M. Verhaak, Jan A. M. Smeitink, Paul de Laat, Mirian C. H. Janssen, José A. E. Custers

**Affiliations:** ^1^ Department of Medical Psychology Radboud University Medical Center, Radboud Institute for Health Sciences, Radboud Center for Mitochondrial Medicine Nijmegen The Netherlands; ^2^ Department of Pediatrics Radboud university medical center, Radboud Institute for Molecular Life Sciences, Radboud Center for Mitochondrial Medicine Nijmegen The Netherlands; ^3^ Department of Pediatrics Franciscus Gasthuis & Vlietland Rotterdam The Netherlands; ^4^ Department of Internal Medicine Radboud university medical center, Radboud Institute for Molecular Life Sciences, Radboud Center for Mitochondrial Medicine Nijmegen The Netherlands

**Keywords:** fatigue, longitudinal, mental health, mitochondrial disease, mtDNA 3243A>G variant, quality of life

## Abstract

Severe fatigue is a common complaint in patients with primary mitochondrial disease. However, less is known about the course of fatigue over time. This longitudinal observational cohort study of patients with the mitochondrial DNA 3243 A>G variant explored trajectories of fatigue over 2 years, and characteristics of patients within these fatigue trajectories. Fifty‐three adult patients treated at the Radboud University Medical Center Nijmegen were included. The majority of the patients reported consistent, severe fatigue (41%), followed by patients with a mixed pattern of severe and mild fatigue (36%). Then, 23% of patients reported stable mild fatigue levels. Patients with a stable high fatigue trajectory were characterized by higher disease manifestations scores, more clinically relevant mental health symptoms, and lower psychosocial functioning and quality of life compared to patients reporting stable low fatigue levels. Fatigue at baseline and disease manifestation scores predicted fatigue severity at the 2‐year assessment (57% explained variance). This study demonstrates that severe fatigue is a common and stable complaint in the majority of patients. Clinicians should be aware of severe fatigue in patients with moderate to severe disease manifestation scores on the Newcastle Mitochondrial Disease Scale, the high prevalence of clinically relevant mental health symptoms and overall impact on quality of life in these patients. Screening of fatigue and psychosocial variables will guide suitable individualized treatment to improve the quality of life.

**Synopsis**

The majority of patients with primary mitochondrial disease reported stable, severe fatigue over a 2‐year period, and were characterized by more severe disease manifestation and experienced lower psychosocial functioning and quality of life than patients with stable mild fatigue.

## 1 INTRODUCTION

Primary mitochondrial diseases (PMDs) are heterogenous, hereditary diseases affecting the oxidative phosphorylation system.^1^ Defects in this system can be caused by mutations in the nuclear or mitochondrial DNA (mtDNA).^2^ Patients with PMD can experience symptoms in any organ or tissue. PMDs are often multisystemic, but mono‐system involvement has been reported. Most affected are high energy depending organs such as the brain, eyes, heart, skeletal muscles, and kidneys. PMDs are generally progressive, though the disease course often remains unpredictable.^3^, ^4^

A common mutation associated with PMDs is the transfer RNA of leucine (UUR) 3243 A>G variant. The variant is associated with two classical mitochondrial syndromes and mixed phenotypes. It can cause Mitochondrial Encephalomyopathy, Lactic Acidosis, and Stroke‐like episodes (MELAS) syndrome, characterized by symptoms such as epilepsy, elevated lactic acid levels, stroke‐like episodes, and exercise intolerance. Psychiatric symptoms are also common.^3^, ^5^ The same genetic variant can also cause Maternally Inherited Diabetes and Deafness (MIDD), characterized by early onset, sensorineural hearing loss, and a deficit in the insulin secretion. The clinical features of patients with the mtDNA 3243 A>G variant are not limited to these syndromes. Patients can experience a variety of clinical features and symptoms can change over time.^3^, ^6^

Fatigue is a common complaint in patients with PMD, irrespective of the involved genetic variation. Percentages of patients with severe perceived fatigue range between 60 and 100%.^7^, ^8^, ^9^, ^10^, ^11^ Perceived fatigue can be characterized as “*an overwhelming sense of tiredness*, *lack of energy or feeling of exhaustion associated with impaired physical and/or cognitive functioning*.”^12^ Disease severity and mental health symptoms were associated with fatigue severity in several studies. More severely affected patients with PMD experienced higher fatigue severity.^7^, ^8^, ^11^ Furthermore, depression and anxiety symptoms were reported by 20–62% of patients with PMD.^8^, ^10^, ^11^

It is yet unclear how the course of fatigue in patients with PMD develops over time. Studies in other progressive diseases such as multiple sclerosis (MS), amyotrophic lateral sclerosis (ALS) and incurable cancer showed fatigue levels remained largely stable,^13^, ^14^, ^15^, ^16^ and depressive and anxiety symptoms were highly common.^17^, ^18^ The influence of disease‐related and psychosocial variables on the course of fatigue varies between studies,^13^, ^14^, ^16^ and these generic factors can influence fatigue irrespective of the disease.^19^

In patients with PMD, knowledge about fatigue severity over time and the relationship with disease‐related and psychosocial factors is needed to further improve patient care and guide future research. Investigating trajectories of fatigue in patients with PMD and associated patient characteristics can help identify patients at risk for (consistent) severe fatigue. The present study will investigate trajectories of fatigue over a 2‐year period in patients with PMD due to the m.3243A > G variant. Disease and psychosocial characteristics will be described and compared for patients in each fatigue trajectory. Furthermore, predictors of fatigue trajectories and fatigue severity at 2‐year follow‐up will be explored.

## 2 METHODS

### 2.1 Participants

Adult patients with PMD due to m.3243A > G variant under care in the Radboud Center for Mitochondrial Medicine, Radboud University Medical Center, and carrier relatives were invited to participate in a longitudinal observational cohort study approved by the local ethical committee (NL32683.091.10).^20^ The present study uses available data from an ongoing cohort study, which was collected in 2014 and 2016.^11^, ^21^

### 2.2 Procedure

Patients were invited to participate in this study by their physician during clinical consultations. After informed consent, patients were invited to fill out online questionnaires focusing on fatigue, mental health, attitude toward the disease, daily functioning, and quality of life (QoL). Patients were followed over a 2‐year period, filling out self‐report questionnaires at baseline (T0), 2 weeks (T1, test–retest assessment), 3 months (T2), and 2 years (T3). Disease‐related outcomes were measured separately by their physician during a clinical consultation within the same year of the questionnaire study. This information was not available for five patients. The clinical assessment closest to the year of the study (2014) was used for these patients.

### 2.3 Instruments

#### 2.3.1 Perceived fatigue

Perceived fatigue was assessed with the Checklist Individual Strength (CIS). The CIS consists of 20 items scored on a 7‐point Likert scale. It assesses four dimensions of fatigue: Fatigue Severity (score range 8–56), Concentration (range 5–35), Motivation (range 4–28), and Physical Activity (range 3–21), higher scores represent higher severity. The subscale Fatigue Severity is used to measure perceived fatigue severity, a score of 35 or higher indicates severe fatigue.^22^, ^23^ The CIS has sufficient reliability and validity^24^, ^25^ and has been used in patients with PMD in previous studies.^8^, ^10^

#### 2.3.2 Disease‐related outcomes

*Genotype* was assessed measuring heteroplasmy levels of the m.3243A > G variant in leukocytes and urinary epithelial cells (UEC).^6^, ^26^ Blood heteroplasmy is negatively correlated with age, therefore, age‐adjusted blood heteroplasmy is investigated using the formula described by Grady et al. (2018): age‐adjusted blood level = (heteroplasmy leukocytes)/0.977^(age+12)^.^27^

*Disease manifestation* was assessed using the Newcastle Mitochondrial Disease Scale (NMDAS), a validated instrument to monitor the clinical expression/progression of PMD over time.^28^ The clinical expression is measured in three sections: (1) Current Functioning (10 items); (2) System Specific Involvement (9 items); and (3) Current Clinical Assessment (10 items). Items are scored from 0 (no involvement) to 5 (severe involvement). The total score on each section was divided into categories as described by Parikh et al. (2019): no clinical manifestation (0), mild (1–5), moderate (6–20), and severe clinical manifestation (≥21).^8^ Details of the assessment and inter‐rater reliability have been described previously by de Laat et al.^20^

#### 2.3.3 Psychosocial outcomes

The Hospital Anxiety and Depression Scale (HADS) measures symptoms of anxiety, depression, and general distress.^29^, ^30^ It was designed for patients with a medical condition, controlling for physical aspects of a medical disease that may confound with symptoms of depression or anxiety. The HADS consists of two subscales, Depression and Anxiety (both seven items). Scores range between 0 and 21, a score of 8 or higher suggests clinically relevant symptoms. The total score represents general distress (range 0–42). The Dutch translation showed good reliability.^29^

Beck's Depression Inventory for Primary Care (BDI‐PC) was used to measure the severity of depressive symptoms.^31^, ^32^ This questionnaire assesses the cognitive and affective aspects of depressive symptoms without somatic‐related items. It consists of seven items, with a total score ranging between 0 and 21. A score of four and higher suggests the presence of a possible major depressive disorder. The reliability in our sample was acceptable with a Cronbach's alpha of.73.

The Illness Cognitions Questionnaire was used to measure illness‐related cognitions, and shows the patients' reevaluation regarding their disease. This study describes outcomes on helplessness and acceptance. Both subscales consist of six items, scores range between 6 and 24 on each subscale.^33^, ^34^

Self‐efficacy was measured with the self‐efficacy questionnaire SE‐28 which assesses the level of control the patient feels regarding their disease and symptoms using seven items. Total scores range between 7 and 28, higher scores reflect a higher self‐efficacy.^35^, ^36^

Aspects of social functioning and support were measured with subscales related to social functioning of the Inventory for Social Reliance (ISR), Sickness Impact Profile (SIP), and RAND‐36. The ISR subscale Perceived Support consists of five items scored on a 4‐point Likert scale. The total score ranges between 5 and 20, higher scores reflect more perceived support.^37^, ^38^, ^39^

Functional impairment due to illness was measured with the SIP. This questionnaire provides information about changes in everyday activities due to sickness.^40^, ^41^ We included 86 items measuring the following subscales: Social Interactions (range 0–1450), Sleep and Rest (range 0–499), Home Management (range 0–668), Mobility (range 0–719), Ambulation (range 0–842), Alertness behavior (range 0–777), Work (range 0–783), Recreation, and Pastime (range 0–422). A score of 0 represents good health, higher scores represent poor health or physical and behavioral impairments due to illness. The Dutch version of the SIP shows good reliability.^42^

QoL was measured with the RAND‐36, assessing the following dimensions: Physical Functioning (range 10–30), Social Functioning (range 2–10), Role Limitations due to Physical Health (range 4–8), and Emotional Problems (range 3–6), Emotional Well‐being (range 5–30), Energy/Fatigue (range 4–24), Pain (range 11–60), General Health (range 5–25), and Health Change (range 1–5). The Dutch version shows good reliability and sufficient construct validity.^43^, ^44^

### 2.4 Data analyses

#### 2.4.1 Classification of the fatigue trajectories

Patients were included when they completed at least two assessments, including the 2‐year assessment (T3). Patients were divided over three groups based on the validated cut‐off score of 35 on the CIS‐Fatigue Severity, representing severe perceived fatigue.^22^, ^23^ Individual fluctuations can be small or large in all groups, though the possible range of scores is larger in the fluctuating group.

Three fatigue trajectories over a 2‐year period were explored based on the completed assessments: (1) stable low fatigue levels: patients in this group reported scores of 34 and lower on the CIS‐Fatigue Severity at all assessments (range 8–34); (2) stable high fatigue trajectory: patients reported scores of 35 or higher on the CIS‐Fatigue Severity at all assessments (range 35–56); (3) fluctuating fatigue trajectory: patients in this group reported scores fluctuating below and above the cut‐off score of 35, from high to low and vice versa, on the CIS‐Fatigue Severity at the completed assessments (range 8–56).

### 2.5 Statistical analyses

Data analysis was performed using SPSS version 25. Descriptive statistics were used to classify fatigue trajectories and to describe patient characteristics in each group. Differences between the fatigue trajectory groups in reported fatigue on the CIS was investigated using one‐way ANOVAs (*α* of 0.05). Pairwise comparison was done using Tukey's HSD test, or Games–Howell test when equal variances cannot be assumed. Baseline characteristics related to the disease manifestation and psychosocial outcomes were compared between patients in different fatigue trajectories using the nonparametric Kruskal–Wallis ANOVA (*α* of .05), because the assumption of normality was violated for most subscales. The Mann–Whitney *U* test was used as pairwise comparison, with adjusted significance scores using the Bonferroni correction. In addition, corrections for multiple testing were made using the Bonferroni correction on the SIP (*α* = 0.006) and RAND‐36 (*α* = 0.005).

To explore individual changes in fatigue severity over time, relative and absolute change scores were calculated for the CIS‐Fatigue Severity between each assessment. To identify possible disease‐related predictors of fatigue trajectories, a multinomial logistical regression analysis was performed. In this analysis, categorized scores on the subscales of the NMDAS were used (no, mild, moderate, and severe clinical manifestation). Predictors of fatigue severity at 2‐year follow‐up were explored using a hierarchical multiple regression analysis. Variables for the regression analyses were chosen based on literature in other chronic illnesses. All entered variables were correlated, no multicollinearity was present.

## 3 RESULTS

### 3.1 Sample characteristics

A total of 72 out of 122 patients (59%) responded and completed the baseline assessment, 69% responded at 2 weeks, 67% at 3 months and 74% at 2‐year follow‐up. In total, 53 participants completed the CIS‐Fatigue Severity at least twice, including the assessment at 2‐year follow‐up. In total, 35 pedigrees were included. Ten families had two to four family members participating in this study, twenty‐five participants had no other family members participating in the study. Demographic characteristics of the patients are summarized in Table 1 according to fatigue trajectory, including mean (*M*) and standard deviations (*SD*) on disease characteristics. Overall, more female patients with the m.3243A > G variant participated in the study than male patients (3.3:1). Asymptomatic carriers had a total NMDAS score of six or lower in our sample. Nonresponders and noncompleters did not significantly differ from the included participants on age, sex, or NMDAS scores. Furthermore, noncompleters scored similar on the CIS‐Fatigue Severity compared with participants that completed at least two assessments (*M* = 38.97, *SD* = 12.47, *M* = 37.58, *SD* = 12.54, respectively). The demographic questionnaire was completed by 42 participants.

**TABLE 1:** Demographics and patient characteristics for each fatigue trajectory and total sample at baseline

	Stable low fatigue (*n* = 12)	Fluctuating fatigue (*n* = 19)	Stable high fatigue (*n* = 22)	Total	*n*
Age (*M*, *SD)*	51.47 (13.70)	43.69 (11.23)	45.12 (14.52)	46.05 (13.33)	53
Sex*: female, *n* (%)	10 (83%)	11 (58%)	20 (91%)	41 (77%)	53
Married/living with partner, *n* (%)	9 (90%)	9 (56%)	12 (75%)	30 (71%)	42
Has children, *n* (%)	9 (92%)	9 (56%)	11 (69%)	29 (69%)	42
**Educational level, *n* (%)**					42
Primary	‐	2 (11%)	3 (14%)	5 (9%)	
Secondary	9 (75%)	10 (53%)	12 (55%)	31 (58%)	
Tertiary	3 (25%)	7 (37%)	7 (32%)	17 (32%)	
**Work status, *n* (%)**					42
Payed	8 (80%)	8 (50%)	5 (31%)	21 (50%)	
Student	‐	1 (6%)	‐	1 (2%)	
Volunteer	‐	2 (13%)	4 (25%)	6 (14%)	
**Clinical phenotype, *n* (%)**					53
MELAS	0	1 (5%)	2 (9%)	3 (6%)	
MIDD	4 (33%)	9 (47%)	12 (55%)	25 (47%)	
Asymptomatic carrier	5 (42%)	4 (21%)	0	9 (17%)	
Other	3 (25%)	5 (26%)	8 (36%)	16 (30%)	
**NMDAS, *M* (*SD*)**					53
Current functioning*	4.50 (4.34)	6.89 (9.70)	10.18 (7.72)	7.72 (7.69)	
System specific involvement*	3.58 (3.80)	5.32 (5.53)	9.23 (6.21)	6.55 (5.90)	
Current clinical assessment	2.42 (3.29)	3.58 (6.79)	4.14 (3.68)	3.55 (4.92)	
Total score*	10.50 (10.77)	15.79 (21.34)	23.55 (14.70)	17.81 (17.24)	
**Heteroplasmy, *M* (*SD*)**					53
Age‐adjusted leukocytes	45.41 (31.49)	55.22 (35.00)	76.97 (57.67)	62.03 (46.39)	
UEC	41.17 (20.40)	54.95 (29.25)	47.77 (20.43)	48.85 (24.09)	

*Note*: Mean (*M*) and standard deviations (*SD*) are shown on age, NMDAS, and heteroplasmy. Number of participants (*n*) and percentage (%) are provided on demographic variables including clinical phenotype.

Abbreviations: MELAS, Mitochondrial Encephalomyopathy, Lactic Acidosis, and Stroke‐like episodes syndrome; MIDD, Maternally Inherited Diabetes and Deafness; NMDAS, Newcastle Mitochondrial Disease Scale; UEC, urinary epithelial cells.

* Significant difference between groups (<0.05).

### 3.2 Characteristics of fatigue trajectories

**FIGURE 1:**
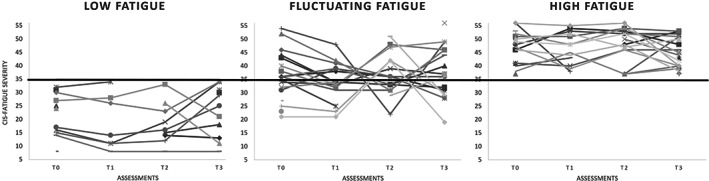
Individual fatigue course of all participants visualized for each fatigue trajectory. Scores reported on T0 (baseline), T1 (2 weeks), T2 (3 months), and T3 (2 years) on the completed assessments by each participant. The cut‐off score (35) is visualized with a black horizontal line through each fatigue trajectory group.

Most patients were classified in the stable high fatigue trajectory (41.5%), followed by fluctuating fatigue (35.9%) and stable low fatigue (22.6%). Fatigue severity differed significantly between groups at T0, T1, T2, and T3 (*p* < 0.05). The course of fatigue of all participants in each group is visualized in Figure 1. Demographics for each fatigue trajectory are described in Table 1.

There were significant differences in distribution of sex between groups (*χ* ^2^ = 6.66, *p* = 0.038). More female patients were present in the stable high fatigue group (91%) compared with the fluctuating fatigue group (58%). No differences in age were observed between groups. Disease‐related outcomes showed that groups differed significantly on Current Functioning (*H* = 8.429, *p* = 0.015), System Specific Involvement (*H* = 12.571, *p* = 0.002), and total score (*H* = 11.883, *p* = 0.003) of the NMDAS. On Current Functioning, patients in the stable low fatigue group scores significantly lower than patients with stable high fatigue. On System Specific Involvement and the total score, patients with a stable high fatigue trajectory scored significantly worse than patients with stable low fatigue and fluctuating fatigue (*p* < 0.05). Though not significant, a similar distribution of the scores was seen on Current Clinical Assessment: patients with a low fatigue trajectory scored lowest, the highest scores on the NMDAS were reported by the group with stable high fatigue. Regarding heteroplasmy levels, no significant differences between groups were found in age‐adjusted leukocytes or UEC levels (*p* > 0.05). However, a trend was visible based on the mean scores on age‐adjusted leukocytes, patients reporting stable low fatigue showed lower heteroplasmy levels, and the stable high fatigue group showed highest heteroplasmy levels. Data on the clinical phenotype suggested differences between groups. In the stable low fatigue trajectory, 42% of the group consisted of asymptomatic carriers, whereas no carriers were present in the stable high fatigue group.

Within‐subject fluctuations in fatigue severity were explored using absolute and relative change scores of fatigue severity between assessments. Fluctuations in fatigue could be positive or negative. The change scores, including the range, are shown in Supplementary Tables 1 and 2. Additional domains of fatigue measured with the CIS at baseline showed patients with low fatigue scored significantly better on concentration than patients with both fluctuating and stable high fatigue (*p* < 0.05). On motivation and physical activity, patients with high fatigue scored significantly worse than patients with stable low fatigue (*p* < 0.01).

### 3.3 Psychosocial outcomes

A detailed overview of outcomes and differences between groups on fatigue and psychosocial outcomes at baseline assessment are reported in Table 2.

**TABLE 2:** Outcomes on disease‐related and psychosocial variables and differences between the stable low, fluctuating and stable high fatigue trajectories (*N* = 53)

Questionnaires—subscales (range of scores)	Stable low fatigue *M* (*SD*)	Fluctuating fatigue *M* (*SD*)	Stable high fatigue *M* (*SD*)	Total *M* (*SD*)	*F*, *p*‐value
CIS* (*M*, *SD*)					
Fatigue severity (range 8–56)	21.33 (7.82)	37.05 (9.60)	46.91 (5.98)	37.58 (12.54)	*F*(2,50) = 41.25, *p* < .001^ABC^
Concentration (range 5–35)	11.08 (4.42)	17.79 (7.60)	22.55 (8.22)	18.25 (8.43)	*F*(2,32.92) = 14.60, *p* < .001^AB^
Motivation (range 4–28)	10.50 (5.39)	15.00 (5.92)	17.05 (5.19)	14.83 (5.96)	*F*(2,50) = 5.51, *p* = .007^B^
Physical activity (range 3–21)	7.00 (3.49)	10.95 (5.48)	14.59 (4.92)	11.57 (5.62)	*F*(2,50) = 9.70, *p* < .001^B^

*Psychosocial variables*	*M* (*SD*)	*M* (*SD*)	*M* (*SD*)	*M* (*SD*)	*H*, *p*‐value
BDI‐PC* (range 0–21)	0.58 (1.17)	2.21 (2.04)	3.73 (2.90)	2.47 (2.57)	*H* = 14.834, *p* = .001^AB^
HADS*					
Total (range 0–42)	4.42 (5.11)	9.16 (6.46)	14.45 (6.05)	10.28 (7.11)	*H* = 17.262, *p* < .001^B^
Anxiety (range 0–21)	2.25 (2.30)	5.16 (3.91)	7.09 (3.29)	5.30 (3.78)	*H* = 15.045, *p* = .001^B^
Depression (range 0–42)	2.17 (3.66)	4.00 (3.40)	7.36 (3.97)	4.98 (4.22)	*H* = 16.456, *p* < .001^BC^
**ICQ**					
Helplessness* (range 6–24)	8.33 (3.03)	10.16 (3.44)	14.50 (3.57)	11.55 (4.24)	*H* = 19.762, *p* < .001^BC^
Acceptance** (range 6–24)	18.42 (6.01)	18.68 (3.87)	14.00 (3.96)	16.68 (4.94)	*H* = 11.893, *p* = .003^BC^
SE‐28** (range 7–28)	20.50 (3.42)	19.89 (3.11)	15.45 (3.50)	18.19 (4.03)	*H* = 18.644, *p* < 0.001
ISR**					
Perceived support (range 5–20)	18.58 (2.15)	17.16 (3.83)	15.64 (3.70)	16.85 (3.60)	*H* = 4.795, *p* = 0.091 (ns)
SIP*					
Social interactions (range 0–1450)	52.25 (85.55)	106.74 (96.75)	210.77 (174.21)	137.58 (145.83)	*H* = 10.571, *p* = .005^B^
Sleep and rest (range 0–499)	14.17 (25.79)	47.95 (43.48)	82.23 (65.97)	54.53 (57.17)	*H* = 12.072, *p* = .002^B^
Home management (range 0–668)	19.08 (38.26)	77.26 (59.44)	137.95 (99.23)	89.28 (87.76)	*H* = 15.781, *p* < .001^B^
Mobility (range 0–719)	3.42 (11.84)	21.95 (46.37)	86.77 (141.61)	44.66 (101.01)	*H* = 8.297, *p* = 0.016 (ns)
Ambulation (range 0–842)	28.67 (99.30)	32.00 (41.91)	101.64 (95.22)	60.15 (87.19)	*H* = 11.109, *p* = .004^B^
Alertness behavior (range 0–777)	12.75 (29.78)	138.89 (116.28)	199.23 (183.16)	135.38 (153.67)	*H* = 14.362, *p* = .001^AB^
Work (range 0–783)	22.58 (44.91)	60.26 (82.45)	20.00 (33.73)	35.02 (60.02)	*H* = 3.564, *p* = 0.168 (ns)
Recreation and pastime (range 0–422)	25.75 (48.43)	62.63 (48.07)	126.59 (75.14)	80.93 (72.69)	*H* = 16.473, *p* < .001^BC^
RAND‐36**					
Social functioning (range 2–10)	8.92 (1.16)	7.42 (1.71)	5.95 (1.81)	7.15 (1.99)	*H* = 18.856, *p* < .001^B^
Physical functioning (range 10–30)	27.00 (4.02)	23.47 (5.03)	19.64 (4.32)	22.68 (5.31)	*H* = 18.723, *p* < .001^AB^
Role limitations (physical) (range 4–8)	6.92 (1.68)	5.63 (1.74)	4.45 (0.91)	5.43 (1.70)	*H* = 15.874, *p* < .001^B^
Role limitations (emotional) (range 3–6)	5.67 (0.78)	5.16 (0.96)	4.55 (1.47)	5.02 (1.23)	*H* = 6.248, *p* = 0.044 (ns)
Emotional well‐being (range 5–30)	25.75 (2.49)	23.00 (3.48)	21.05 (3.92)	22.81 (3.88)	*H* = 11.080, *p* = .004^B^
Pain (range 11–60)	51.00 (12.30)	47.21 (10.54)	39.82 (12.35)	45.00 (12.39)	*H* = 6.727, *p* = 0.035 (ns)
Energy/fatigue (range 4–24)	17.08 (3.00)	14.47 (3.49)	11.50 (1.92)	13.83 (3.53)	*H* = 15.340, *p* < .001^BC^
General health (range 5–25)	17.00 (4.69)	15.32 (3.53)	10.36 (2.24)	13.64 (4.38)	*H* = 23.199, *p* < .001^AB^
Health change (range 1–5)	3.00 (0.60)	2.68 (1.00)	2.50 (0.91)	2.68 (0.89)	*H* = 3.179, *p* = 0.204 (ns)

*Note*: Mean (*M*) and standard deviations (*SD*) are provided for the subgroups and total sample for each questionnaire. Test statistics are provided on the one‐way ANOVA on the CIS and Kruskal–Wallis ANOVA for all other questionnaires; non‐significance is shown as (ns). Kruskal–Wallis nonparametric test: *H* was corrected for ties, *df* = 2, *N* = 53 for all analyses, pairwise comparisons used adjusted significance using the Bonferroni correction. Corrections for multiple testing were made using the Bonferroni correction on the SIP (*α* = 0.006) and RAND‐36 (*α* = 0.005). Significance between groups using pairwise comparisons is visualized with ABC in the last column (all *p* < 0.05): ^A^ represents significant difference between stable low fatigue and low/high fatigue; ^B^ represents significant difference between stable low and stable high fatigue; and ^C^ represents significant difference between low/high fatigue and stable high fatigue groups. Single asterisk denotes higher scores on this questionnaire/subscale represent worse functioning and double asterisk denotes higher scores on this questionnaire/subscale reflect better functioning.

Abbreviations: BDI‐PC, Beck's Depression Inventory for Primary Care; CIS, Checklist Individual Strength; HADS, Hospital Anxiety and Depression Scale; ICQ, Illness Cognitions Questionnaire; ISR, Inventory for Social Reliance; SE‐28, self‐efficacy questionnaire; SIP, Sickness Impact Profile.

#### 3.3.1 Stable high versus stable low fatigue trajectories

Patients with stable high fatigue experienced significantly more problems on mental health and psychosocial functioning. Regarding mental health, patients with stable high fatigue scored worse on general distress, anxiety, and depressive symptoms than patients with stable low fatigue (*p* ≤ 0.001). Scores on depressive and/or anxiety symptoms on the HADS indicating the presence of a possible mental disorder (≥8) were more common in patients with stable high fatigue levels (HADS: *χ* ^2^ (2, *N* = 53) = 8.992, *p* = 0.011; BDI‐PC: *χ* ^2^ (2, *N* = 53) = 6.040, *p* = 0.049). At baseline, clinically relevant scores were reported by 59% of patients with stable high fatigue, compared with 8% of patients with low fatigue levels. Subgroup analysis of clinically relevant depressive scores on the BDI‐PC was not significant. The presence of clinically relevant depressive and/or anxiety scores was similar at 2‐year follow‐up (see Supplementary Table 3).

Patients with stable high fatigue reported more negative attitudes toward the disease. On helplessness, acceptance and self‐efficacy, patients with stable high fatigue scored significantly worse compared with patients with stable low fatigue (*p* < 0.05). Patients with stable high fatigue also reported more impairments in all domains of daily life, except work and mobility. Regarding social functioning, severely fatigued patients experienced more impairments related to social interactions and lower satisfaction with their social functioning. Although a similar trend was observed on perceived social support, no significant differences were present. Patients with stable high fatigue also experienced lower QoL, they reported less satisfaction on all domains of the RAND‐36 except on Health Change, Pain, and Role Limitations due to Emotional Problems.

#### 3.3.2 Stable high versus fluctuating fatigue trajectories

Outcomes regarding mental health showed no differences between groups, except depressive scores on the HADS: patients with consistent severe fatigue reported higher depression scores. Clinically relevant depressive and/or anxiety scores were present in 32% of patients with fluctuating fatigue, compared to 59% of patients with stable high fatigue (not significant).

Patients with stable high fatigue experienced more helplessness, less acceptance and lower self‐efficacy toward their disease and its consequences (*p* < 0.005). No significant differences on social functioning were found. Patients with stable high fatigue experienced more impairments in daily life on one domain: recreation and pastime. They also reported less satisfaction regarding their energy levels than patients with fluctuating fatigue.

#### 3.3.3 Fluctuating versus stable low fatigue trajectories

Regarding mental health, patients with stable low fatigue trajectories scored significantly lower on depression measured with the BDI‐PC than patients with fluctuating fatigue (*p* < 0.05). However, no differences were present on distress, depression and anxiety measured with the HADS, or regarding attitudes toward the disease.

No significant differences related to social functioning were present between patients with fluctuating fatigue and stable low fatigue. Patients with fluctuating fatigue reported more impairments related to alertness behavior. QoL was similar between groups, except on physical functioning and general health: patients in the stable low fatigue group scored better than patients in the fluctuating fatigue group.

### 3.4 Predictors of fatigue

Four disease‐related and demographic variables were included in the final model: Current Functioning, System Specific Involvement, and Clinical Functioning of the NMDAS, and sex. The stable high fatigue group was entered as the reference group. The model was statistically significant, *χ* ^2^ = 27.161, *p* = 0.001, *df* = 8. System Specific Involvement and sex were significant predictors within the model. Patients with lower scores on System Specific Involvement were more likely to have a stable low than a stable high fatigue trajectory. Male patients were more likely to have fluctuating fatigue than stable high fatigue (see Table 3).

**TABLE 3:** Results from the multinomial logistical regression analysis on fatigue trajectories

		95% CI for odds ratio		
Stable low^a^	*p*	Lower	Odds ratio	Upper
Intercept	0.067			
Current functioning	0.530	0.14	0.62	2.80
System specific involvement	**0.011**	**0.01**	**0.07**	**0.64**
Clinical functioning	0.231	3.25	3.25	22.36
Sex	0.595	0.32	0.48	7.18

		95% CI for odds ratio		
Fluctuating^a^	*p*	Lower	Odds ratio	Upper
Intercept	0.006			
Current functioning	0.102	0.86	0.33	1.25
System specific involvement	0.697	0.15	0.73	3.53
Clinical functioning	0.776	0.23	0.83	3.03
Sex	**0.012**	**0.01**	**0.07**	0.55

*Note*: Variables in bold were significant predictors (*p* < 0.05).

^a^ Compared to the stable high fatigue group.

Second, five factors were explored as possible predictors of fatigue severity at 2‐year follow‐up. A hierarchical regression analysis was performed. First, baseline fatigue scores were entered, followed by disease‐related variables, age and psychological variables measured at baseline assessment in the following order: NMDAS‐total, age‐adjusted heteroplasmy levels in leukocytes, age, depressive symptoms (HADS), and anxiety symptoms. The final model significantly predicted fatigue severity at 2‐year follow‐up, *F*(6,46) = 11.11, *p* < 0.001, *R* ^*2*^ = 0.592. Fatigue severity at baseline significantly explained 52.3% of the variance (*β* = 0.607, *p* < 0.001), disease manifestation added 4.6% explained variance (*β* = 0.221, *p* < 0.001). All other variables were not significant.

## 4 DISCUSSION

This exploratory study investigated fatigue complaints over a 2‐year period in patients with PMD due to the m.3243A > G variant. Patients were divided into three groups based on their fatigue levels at multiple assessments. Stable high fatigue was most common (41%), followed by patients with fluctuating fatigue and stable low fatigue trajectories (23%). Compared to patients with a stable low fatigue trajectory, patients with stable high fatigue were characterized by worse clinical functioning and more (severe) organ system involvement. In addition, patients with stable high fatigue reported worse psychosocial functioning, including a higher prevalence of mental health symptoms, more functional impairments and lower QoL. Furthermore, the main predictors of fatigue at 2‐year follow‐up were fatigue severity at baseline and disease manifestation on the NMDAS (57% explained variance).

The majority of patients with the m.3243A > G variant reported severe fatigue complaints on at least one assessment (77%). This corresponds with previous studies in patients with PMD.^7^, ^8^ The results of the present study suggest fatigue remains stable for most patients, scoring either consistently below or above the cut‐off score indicating mild or severe fatigue complaints. Higher fatigue levels at the 2‐year assessment were predicted by more severe fatigue at baseline, suggesting severe fatigue is rather consistent and difficult to influence. Though the use of additional, regular assessments may show an increase of patients experiencing fluctuations over time, a relatively stable course of fatigue corresponds with studies in other diseases.^13^, ^14^, ^15^, ^16^, ^45^

Results showed disease manifestation (NMDAS) had a significant contribution to fatigue complaints. A positive correlation between disease manifestation on the NMDAS and fatigue severity in patients with PMD has been reported in multiple studies.^46^ Regarding heteroplasmy, studies in patients with the m.3243A > G variant have reported age‐corrected blood heteroplasmy levels as predictor of both disease burden and stroke‐like episodes.^27^, ^47^ However, age‐adjusted heteroplasmy in leukocytes was no significant predictor of fatigue in our study nor were significant differences reported between groups. However, a trend was visible with patients in the stable high fatigue trajectory had higher age‐adjusted heteroplasmy levels than patients with stable low fatigue. The impact of PMD on fatigue is complex. Biological mechanisms including cytokines, mitochondrial dysfunction and genes, but also psychological variables such as depression have been linked to fatigue.^48^ Disease‐related and generic variables have been reported as predictors of fatigue severity in other diseases such as MS, ALS, and PD.^14^, ^49^, ^50^, ^51^, ^52^ Wijenberg et al. described a path analysis of MS‐related fatigue in a cross‐sectional study, in which depression and physical disability were directly related to fatigue whereas disease severity had an indirect effect on fatigue.^53^

Our study showed a sex difference regarding fatigue trajectories. Male patients were more likely to report fluctuating fatigue (42%) than stable high fatigue (8%). This is in contrast with other studies in PMD in which no correlation between sex and fatigue has been reported.^7^, ^8^ Studies in other diseases and the general population do suggest women are more likely to experience severe fatigue than men.^54^, ^55^, ^56^ Another possibility is a type 1 error, our finding could be caused by the small group of male participants in our study (23% male) or the small sample size in the multinomial regression analysis.

Results on mental health showed clinically relevant depressive and anxiety symptoms were present in 25 and 30% of the patients, respectively. This corresponds with other studies in patients with PMD.^8^, ^9^, ^10^ This study highlighted patients with severe fatigue were more likely to experience mental health problems than patients experiencing stable mild fatigue (59 vs. 8%). Mitochondrial dysfunction may be causally related to the high prevalence of these symptoms. Research in patients with psychiatric disorders showed dysfunction of mitochondria in major depression and anxiety disorders.^57^, ^58^ However, depression and anxiety are common in patients with a variety of chronic illnesses,^13^, ^17^, ^18^, ^48^ suggesting the involvement of disease‐generic biological (and psychological) mechanisms. Another possible explanation is depressive or anxiety symptoms may affect self‐reported fatigue complaints. The medical team treating patients with PMDs should be aware of the high prevalence of both fatigue and mental health symptoms and the negative impact on QoL. Psychosocial interventions could support patients in dealing with the impact of the disease on daily life.

These findings have implications for clinical practice. The present study showed a higher burden in patients with severe fatigue complaints affecting many domains of daily life: more mental health problems, more functional impairments, and lower QoL than patients with mild fatigue. Screening patients with severe fatigue complaints additionally on psychosocial functioning is important to provide suitable (multidisciplinary) interventions to improve QoL. The clinical guidelines by Parikh et al. recommended to evaluate treatable etiologies (e.g., anemia, nutritional deficiencies, endocrinopathy) as treatment of fatigue complaints in patients with PMD.^59^ In patient care, patients often receive one or more supportive interventions targeting fatigue‐related complaints (including dietary, physical, occupational, and psychological therapy). However, few studies exist on the effectiveness of these interventions on fatigue severity in patients with PMD. Our findings suggest fatigue, disease severity, and psychosocial outcomes are closely related. Future research should investigate how fatigue can be treated most effectively.

A strength of this study is its longitudinal design. Fatigue was measured over a 2‐year period, keeping into account both short‐term (3 months) and long‐term (2‐year) changes in fatigue levels. Differences regarding mental health and daily functioning in relation to fatigue severity over time were highlighted. Furthermore, exploring predictors of fatigue severity at 2‐year follow‐up and the fatigue trajectories provides information on possible risk factors of consistent, severe fatigue. A limitation of this study is the sample size. Although the number of included patients was respectable given the studied group, it affected the possibilities and power of analyses comparing the fatigue trajectories. Caution is needed interpreting the results of this study, especially with regard to the multinomial regression analysis. Due to the explorative nature of this study, no correction for multiple testing was done. This may result in an overestimation of the significant results.^60^ More research is needed to replicate our findings and investigate fatigue trajectories in patients with similar and other mitochondrial syndromes and genetic variants. To improve statistical validity of the results, longitudinal studies with larger samples and regular assessments are needed to allow additional statistically driven exploration of fatigue trajectories using, for example, latent class growth analysis.^61^, ^62^, ^63^

The results of our study highlight the importance of longitudinal studies capturing the natural course of the disease and domains related to the patients' QoL, including information on disease manifestation (e.g., age of onset, genetic variant, pedigree, symptomology, clinical functioning, and progression); fatigue (e.g., perceived fatigue, performance‐related fatigue, cognitive/physical fatigue); psychosocial variables (e.g., mental disorders, daily stressors/life events, clinical evaluation); and daily functioning. Second, studies investigating the effectiveness of (multidisciplinary) interventions targeting fatigue and associated problems in psychosocial and daily functioning are important. Investigating both risk factors and interventions may provide tools to prevent, reduce, or delay severe, long‐term fatigue complaints in patients with PMD and improve their QoL.

In conclusion, fatigue is a common and relatively stable complaint in patients with m.3243A > G variant over a 2‐year period. Patients with consistent, severe fatigue complaints experienced lower psychosocial functioning, more functional impairments and lower QoL than patients with low fatigue trajectories. This study highlights the importance of screening for fatigue complaints and psychosocial functioning in patients with PMD. More research regarding the course of fatigue over time and identifying risk factors of severe fatigue is needed. The effectiveness of (multidisciplinary) interventions targeting fatigue complaints that have been shown effective in patients with other chronic conditions should be investigated on decreasing fatigue complaints, and comorbid mental health complaints and/or functional impairments. This can improve the QoL of patients with PMD, while awaiting treatment options that may slow, stop, or cure the disease.

### AUTHOR CONTRIBUTIONS

Inge‐Lot Klein was involved in the data analysis and interpretation and writing the article. Paul de Laat, Mirian C. H. Janssen, and Jan A. M. Smeitink participated in conception and design of the study, supervised collection of clinical data, and critically commented on the manuscript. Christianne M. Verhaak and José A. E. Custers were involved in conception and design of the study, data collection, analysis and interpretation, and critically reviewed the manuscript. All authors read and approved the final version of the manuscript.

## CONFLICT OF INTEREST

This study was not industry sponsored. Inge‐Lot Klein, Dr. Christianne M. Verhaak, Dr. Paul de Laat, Dr. Mirian C. H. Janssen, and Dr. José A. E. Custers declare that they do not have any conflict of interest. Prof. Jan A. M. Smeitink is the founding CEO of Khondrion BV.

## ETHICS STATEMENT

This study received approval by a regional human research ethics committee (CMO Nijmegen‐Arnhem), the Netherlands (2010/183; NL32683.091.10).

## INFORMED CONSENT

All procedures followed were in accordance with the ethical standards of the responsible committee on human experimentation (institutional and national) and with the Helsinki Declaration of 1975, as revised in 2000 (5). Written informed consent was obtained from all patients for being included in the study.

## ANIMAL RIGHTS

This article does not contain any studies with animal subjects performed by any of the authors.

## Supporting information


**Supplementary Table 1** Absolute and relative change scores of fatigue severity (CIS) between assessments.
**Supplementary Table 2** Direction of changes on the CIS‐fatigue severity on all assessments
**Supplementary Table 3** Presence of clinically relevant levels of depressive symptoms (BDI‐PC and HADS) and anxiety symptoms (HADS) in patients with stable low, fluctuating or stable high fatigue at baseline and 2‐year follow‐up. Compliance with Ethics Guidelines

## Data Availability

The dataset used during the current study is available from the corresponding author on reasonable request.
