# Supplementary material for: Identifying trajectories of fatigue in patients with primary mitochondrial disease due to the m.3243A > G variant
Source: J Inherit Metab Dis. 2022 Aug 24;45(6):1130–42. doi: 10.1002/jimd.12546 (PMC9805089; doi:10.1002/jimd.12546)
Supplement: Supplementary file 1 — Supplementary Table 1 Absolute and relative change scores of fatigue severity (CIS) between assessments. Supplementary Table 2 Direction of changes on the CIS‐fatigue severity on all assessments Supplementary Table 3 Presence of clinically relevant levels of depressive symptoms (BDI‐PC and HADS) and anxiety symptoms (HADS) in patients with stable low, fluctuating or stable high fatigue at baseline and 2‐year follow‐up. Compliance with Ethics Guidelines [file JIMD-45-1130-s001.pdf]

Supplementary Table 1

Absolute and relative change scores of fatigue severity (CIS) between assessments.

**Group**	**Absolute change**			**Relative change**								
	T0 – T1 (n=37) *(test-retest)*	T0 – T2 (n=39)	T2 – T3 (n=42)	T0 – T1 *(test-retest)*			T0 – T2			T2 – T3		
	M (*SD*)	*M* (*SD*)	*M* (*SD*)	*M* (*SD*)	Min	Max	*M* (*SD*)	Min	Max	*M* (*SD*)	Min	Max
**Low**	3.57 (1.72)	4.10 (3.07)	8.30 (6.72)	-2.71 (3.04)	-6	+2	-1.90 (4.92)	-10	6	2.70 (10.66)	-15	+17
**Mixed**	5.38 (3.42)	8.73 (2.25)	9.94 (8.47)	-3.50 (5.43)	-10	+8	-1.67 (13.91)	-32	21	0.56 (13.30)	-23	+27
**High**	4.36 (4.78)	2.43 (1.95)	5.69 (4.91)	-0.79 (6.53)	-18	+8	1.29 (2.89)	-3	7	-2.31 (7.27)	-16	+14
**Total**	4.65 (3.76)	6.00 (6.76)	7.93 (6.97)	-2.32 (5.55)	-18	+8	-0.67 (9.06)	-32	21	-0.02 (10.63)	-23	+27

Supplementary Table 2

Direction of changes on the CIS-fatigue severity on all assessments

**Group**	**No change (*n*, %)**	**Decreasing scores (*n*, %)**	**Fluctuating scores (*n*, %)**	**Increasing scores (*n*, %)**
**Low**	2 (17%)	2 (17%)	8 (67%)	0
**Mixed**	0	3 (16%)	13 (68%)	3 (16%)
**High**	0	5 (23%)	14 (64%)	3 (14%)

Note: number of participants (n) and percentage (%) in each fatigue trajectory group reporting either no change, decreasing, fluctuating or increasing scores on all completed assessments of the CIS subscale fatigue severity.

Supplementary Table 3

Presence of clinically relevant levels of depressive symptoms (BDI-PC and HADS) and anxiety symptoms (HADS) in patients with stable low, fluctuating or stable high fatigue at baseline and two-year follow-up.

**Group**	**Mental health symptoms**	**T0**	**T3**
Stable low fatigue *n*=12	Clinically relevant depressive symptoms BDI-PC	8%	n/a[a]
	Clinically relevant depressive symptoms HADS	8%	8%
	Clinically relevant anxiety symptoms HADS	0%	8%
	No elevated symptoms HADS	92%	83%
Mixed low/high fatigue *n*=19	Clinically relevant depressive symptoms BDI-PC	21%	n/a[a]
	Clinically relevant depressive symptoms HADS	21%	10%
	Clinically relevant anxiety symptoms HADS	27%	21%
	No elevated symptoms HADS	68%	74%
Stable high fatigue *n*=22	Clinically relevant depressive symptoms BDI-PC	45%	n/a[a]
	Clinically relevant depressive symptoms HADS	36%	45%
	Clinically relevant anxiety symptoms HADS	23%	41%
	No elevated symptoms HADS	41%	41%

At T0, differences between groups were present on the HADS: χ^2 (2, N=53) = 8.992, p = .011, and BDI-PC: χ^2 (2, N=53) = 6.040, p = .049).

[a] BDI-PC was not measured at the two-year assessment.

Abbreviations: BDI-PC, Beck's Depression Inventory for Primary Care; HADS, Hospital Anxiety and Depression Scale;
